# Microbe-Plant Interactions Targeting Metal Stress: New Dimensions for Bioremediation Applications

**DOI:** 10.3390/jox13020019

**Published:** 2023-06-01

**Authors:** Baljeet Singh Saharan, Twinkle Chaudhary, Balwan Singh Mandal, Dharmender Kumar, Ravinder Kumar, Pardeep Kumar Sadh, Joginder Singh Duhan

**Affiliations:** 1 Department of Microbiology, CCS Haryana Agricultural University, Hisar 125004, India; 2 Department of Animal Biotechnology, Lala Lajpat Rai University of Veterinary and Animal Sciences, Hisar 125004, India; 3 Department of Forestry, CCS Haryana Agricultural University, Hisar 125004, India; 4 Department of Biotechnology, Deenbandhu Chhotu Ram University of Science and Technology, Murthal 131039, India; dkbiology@gmail.com; 5 Department of Biotechnology, Chaudhary Devi Lal University, Sirsa 125055, India; rsulakh@gmail.com (R.K.);

**Keywords:** bioremediation, heavy metal, phytoremediation, nanoparticles, computational tools

## Abstract

In the age of industrialization, numerous non-biodegradable pollutants like plastics, HMs, polychlorinated biphenyls, and various agrochemicals are a serious concern. These harmful toxic compounds pose a serious threat to food security because they enter the food chain through agricultural land and water. Physical and chemical techniques are used to remove HMs from contaminated soil. Microbial-metal interaction, a novel but underutilized strategy, might be used to lessen the stress caused by metals on plants. For reclaiming areas with high levels of heavy metal contamination, bioremediation is effective and environmentally friendly. In this study, the mechanism of action of endophytic bacteria that promote plant growth and survival in polluted soils—known as heavy metal-tolerant plant growth-promoting (HMT-PGP) microorganisms—and their function in the control of plant metal stress are examined. Numerous bacterial species, such as Arthrobacter, Bacillus, Burkholderia, Pseudomonas, and Stenotrophomonas, as well as a few fungi, such as Mucor, Talaromyces, Trichoderma, and Archaea, such as Natrialba and Haloferax, have also been identified as potent bioresources for biological clean-up. In this study, we additionally emphasize the role of plant growth-promoting bacteria (PGPB) in supporting the economical and environmentally friendly bioremediation of heavy hazardous metals. This study also emphasizes future potential and constraints, integrated metabolomics approaches, and the use of nanoparticles in microbial bioremediation for HMs.

## 1. Introduction

Environmental contamination has significantly increased over the last few decades as a result of global industrialization. Mining as well as the final disposal of hazardous metal effluents and metal chelates from steel businesses, battery manufacturers, and thermal power plants have been blamed for the deterioration of water and soil quality [1]. Metals are necessary for biological processes in plants and animals, but large amounts can disrupt metabolic processes. Through reducing photosynthetic activity, plant mineral nutrition, and vital enzyme activity, toxic HMs like lead (Pb), uranium (Ur), nickel (Ni), silver (Ag), and chromium (Cr) can hinder plant growth [2]. The hexavalent version of chromium is the most dangerous of all these elements. It results from various processes, including those used in the tanning, electroplating, cooling tower, and paint and dye industries. It significantly negatively impacts agricultural productivity, soil fertility, and water quality [3].

Moreover, the development of plants, nutritional absorption, metabolism, and other physiological processes are all hampered by HMs in soils [4]. HMs are generally defined as elements (both metals and metalloids) that are toxic and have an atomic density greater than 6 gcm^−3^. The ores contain oxides of aluminum, antimony, gold, manganese, and selenium, as well as sulfides of arsenic, cobalt, iron, lead, nickel, and silver. When they enter the environment, they continue to be dangerous for a lot longer [5]. Many of these environmental pollutants are carcinogenic for both humans and the ecosystem. As the body absorbs HMs, they build up in the brain, liver, and kidneys [6]. According to projections, heavy metal poisoning will harm more than 150 million km^2^ of China’s agricultural land, causing an estimated 20 billion Chinese yuan in yearly economic losses [7]. Excessive metal toxicity affects plant development and metabolism by inhibiting cytoplasmic enzymes in plant cells and causing oxidative stress to cell structures. High levels of Pb exposure can cause paralysis and a loss of coordination, whereas severe Cd exposure affects internal organs such as the kidney, liver, and cardiac tissues. Chromium concentrations in underground water can reach up to 14 mgL^−1^ and 660–1700 ppmL^−1^ in India [8]. Via Cr residues, Cr dust, and Cr waste-water irrigation, modern agriculture steadily releases Cr into the environment, causing soil contamination that affects human reproduction, quality, and the soil-vegetable system. Furthermore, in the USA, the United States Environmental Protection Agency (EPA) claims that the country generates more than 50 million metric tons of toxic materials yearly, and 275 different chemicals are classified as priority substances that enhance the toxicity level [9]. The first hazardous waste identified on the ATSDR (Agency for Toxic Substances and Disease Registry) list is arsenic, followed by lead, cadmium, cobalt, nickel, and zinc, all of which pose severe risks to human health. Heavy metal contamination case studies have been recently reported in many countries [10]. Rice, for example, is a major source of arsenic pollution in India and Bangladesh, where an estimated 100 million people are at risk of arsenic poisoning [10].

Metal-contaminated soils are cleaned up using a variety of biological, chemical, and physical processes [11]. Environmental pollutants are being removed using bioremediation technology at an accelerated rate since HMs are a practical and cost-effective solution [12]. Moreover, it has been found that these harmful metals build up in the soil and interfere with bacterial populations, metabolic processes, crop development, symbiosis, and yields [13]. Location-specific environmental factors include pH, temperature, oxygen, and moisture availability. They might influence the metal remediation process by preventing the growth of indigenous pollutant-degrading and pollutant-transforming microorganisms [14]. According to reports, the indigenous microbial population of the soil frequently plays vital roles in plant growth regulation, pest control, soil structure maintenance, nutrient recycling, and pollutant transformation [15,16].

Our study focused on using microorganisms as a potential tool for detoxifying HM-contaminated soils. According to a recent study, using more than one live organism produces effective and improved results, opening the door to research on more diverse microbial species for the bioremediation process. The capacity of microbes to degrade pollutants depends on the environmental suitability for their growth and metabolism, including pH, moisture content, and temperature [17,18]. The effects of remediating HMs with the use of nanoparticles on the environment are also covered in this work.

## 2. Effects of HMs on the Environment 

Due to the non-biodegradable nature of HMs, their removal from the various polluted sites has become a severe problem for the entire globe. Metals, *viz.* Co, Ni, Cu, Mo, Fe, and Mn, are necessary in tiny amounts for organismal survival. However, high doses of these metals are toxic to living things [19]. Metals and metalloids, for example, Ni, Cd, Cr, Hg, As, and Se, are hazardous to soil and crop health if their concentration exceeds the ERCLA (Environmental Response Compensation and Liability Act) maximum permitted concentration, which is: Cr (0.02 mg L^−1^), Se (0.009 mg L^−1^), Hg (0.002 mg L^−1^), Ni (0.03 mg L^−1^), As (0.04 mg L^−1^), Cd (0.02 mg L^−1^) [20]. These pollutants are important contributors to life-threatening human degenerative diseases. Laboratory tests have shown that increased amounts of heavy metals harm the respiration process, the ETC (Electron Transport Chain), photosynthesis, and cell division [21]. As a result of the negative effects of these HMs on the environment, coordinated efforts are required to remove them from the environment to maintain the ecosystem [22].

## 3. HMT-PGP (Heavy Metal Tolerant-Plant Growth Promoting) Microbial Mechanisms for Soil Heavy Metal(loid)s Remediation

HMs displace key components in biological molecules by impairing their activities and altering the function and structure of proteins, enzymes, and membrane transporters [23]. Microbial bioremediation refers to microbes’ techniques to remove or detoxify HMs/metalloids from a contaminated location. As a result, they are employed in the agricultural system to treat diverse HM stresses in numerous plants. It is utilized in the bioremediation of HMs in heavily contaminated places to reduce the impact of these pollutants on various life forms [24]. Heavy metal remediation treatments include electro-dialysis and reverse osmosis. It also includes physical treatments such as extraction, stabilization, immobilization, and soil washing [25,26]. Even if these techniques are effective, they are usually costly because of the high energy and chemical reagent requirements and additional harmful end-products [2]. Table 1 illustrates the bioremediation processes aided by several PGP bacteria and associated methods [27]. Microorganisms are important in HM clean-up because they can tolerate metal toxicity in various ways. According to research, HM bioremediation will be more successful if consortia of bacterial strains are used rather than a single strain culture [28,29]. 

**Figure 1:**
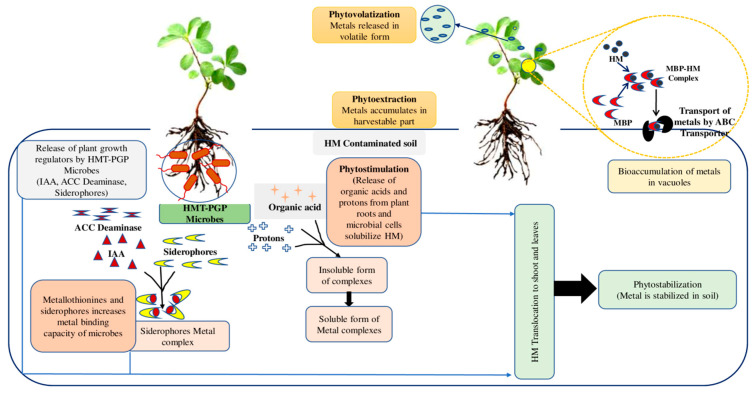
The mechanisms used by HMT-PGP microbes in the remediation of HMs from contaminated soils.

Plant-microbe interaction studies can provide promising solutions for sustainable agriculture. These studies are important in developing bioremediation processes [49]. HMT-PGP bacteria/microorganisms can affect plant development and alter soil physicochemical characteristics to increase metal bioavailability. It results in fast detoxification or removal of HM from the soil. HMT-PGP microorganisms use acidification, complexation, chelation, redox processes, and precipitation to change metal bioavailability in the soil [50]. Four strains were used in a study for the bioremediation of a Pb, Cd, and Cu mixture from contaminated soils: *Viridibacillus arenosi* B-21, *Sporosarcina soli* B-22, *Enterobacter cloacae* KJ-46, and *E. cloacae* KJ-47 [51]. After 48 h, bacterial combinations outperformed single-strain cultures regarding durability and efficacy for HMs remediation, with bioremediation efficiencies of 99% for Pb, 86% for Cd, and 6.6% for Cu. Microbial bioremediation includes mechanisms such as (1) toxic metal sequestration by metallothioneins (MT) and intracellular metal-binding peptides and phytochelatins, as well as compounds like bacterial siderophores and catecholates; (2) modification of metabolic processes to prevent metal uptake; and (3) enzymatic processes that help in the conversion of metals to harmless forms; (4) intracellular concentrations of metals are reduced through precise efflux mechanisms [52]. Figure 1 depicts the processes employed by HMT-PGP microorganisms in the HMs clean-up of polluted soils. Acidic pH levels facilitate HM uptake and sorption in the rhizosphere by increasing the mobility of heavy metal ions. Organic acids produced by these microorganisms also helped sequester soluble metal ions and lower the soil pH [53].

Several studies have revealed that different bacteria and fungi can release organic acids that act as natural HM chelating agents. AMF (arbuscular mycorrhizal fungi) strengthens plants’ adaptability toward climate change. It provides tolerance against stressed agro-climatic conditions, including an unfavorable temperature range, toxic/HMs, salinity, heat, and drought conditions [54]. Potent solubilizers are oxalic acid, gluconic acid, acetic acid, and malic acid [55,56]. Excessive production of organic acids and enzymes in the rhizosphere aids in acidification and electron transfer, resulting in increased metal bioavailability [57]. Changes in exudate component concentrations in the presence of a particular HM can also aid in creating biomarkers [58]. For the transformation of HM into a non-toxic form, the microbial-driven redox process plays an important role. These metal reduction activities of bacteria are aided by outer membrane C-type cytochromes, porin-cytochrome protein complexes (Pcc), and the extracellular electron of MtrABC [53]. Proteins in all these species are up-regulated and aid in HM detoxification in plants. Cr-tolerant *Cellulosimicrobium cellulans* converts Cr^6+^ to non-toxic Cr^3+^ and facilitates its uptake by green chili [59]. *Geobacillus* sp. and *Bacillus* sp. isolated from As-contaminated soil help in the biotransformation of As^3+^ to less toxic As^5+^ [60]. Aside from that, bioaccumulation is vital for detoxification and HM uptake by HMT-PGP bacteria [61].

**Figure 2:**
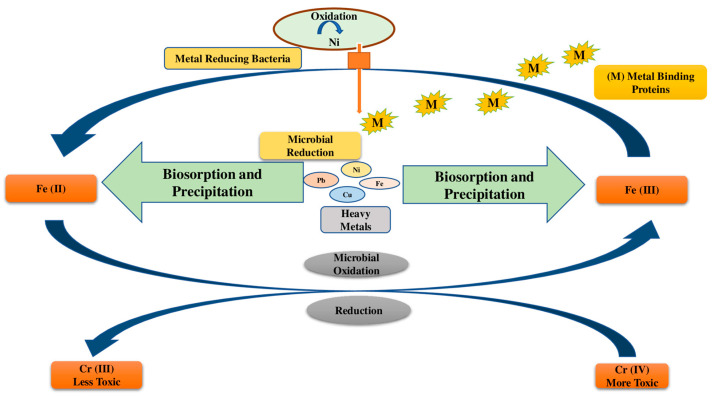
Mechanisms used by microbes for the remediation of HMs in polluted soils are depicted in Figure 2.

Mechanisms used by microbes for the remediation of HMs in polluted soils are depicted in Figure 2. Mainly chelation, coordination, complexation, micro-precipitation, ion exchange, and entrapment occur during biosorption. The cell wall composition and various functional groups such as -OH, -COOH, and -SH have a strong affinity for HMs. However, metallothioneins and glutathione-derived peptides aid in the metal-binding process [62]. 

Fungi, rhizospheric bacteria, and plants produce glutathione peptides and metallothioneins in response to heavy metal stress, which leads to HM buildup in microbial or plant cells. Metallothioneins have a high affinity for Cu, Cd, and Hg due to a potent cysteine group. In one study, the presence of metallothioneins was found to cause a considerable buildup of Pb concentrations in *Bacillus cereus* [63]. The purpose of HMT *Providencia vermicola* strain SJ2A MT-assisted periplasmic Pb sequestration was established by Sharma et al. [64].

The HMT fungus has also been intensively explored for its capacity to detoxify HM by producing MT. However, the expression and synthesis of MT-related genes in HM in mycorrhizal fungi have drawn a lot of interest [65]. After entering the cell, the penultimate stage of HM detoxification is sequestration or compartmentalization into various subcellular organelles. HM vacuolar compartmentation is mainly seen in mycorrhizal fungi. *Glomus intraradices* extra-radical mycelium, renamed *Rhizophagusir regularis*, was found to have vacuolar compartmentalization of Zn, Cu, and Cd [66]. In response to hazardous HM exposure, exopolysaccharide (EPS) synthesis by some PGP bacteria induces biofilm development. Biofilm development improves microbial cell tolerance by forming a protective coating and converting harmful metal ions into non-toxic forms after adsorption [67,68]. It has been demonstrated that EPS produced by rhizobia and other PGP bacteria with a range of anionic groups may sequester various types of HM. The most critical component in such bacterial cells with ion sequestration capacity is exopolysaccharide (EPS). Exopolysaccharide is mainly composed of complex, high-molecular-weight organic macromolecules like polysaccharide, with trace amounts of protein and uronic acid [69]. *Bacillus* spp., *Alcaligenes faecalis*, *Leuconostoc*, *Agrobacterium* spp., *Xanthomonas campestris*, *Zygomonas mobilis*, *Pseudomonas* spp., and *Acetobacter xylinum* are examples of microorganisms that produce exopolysaccharides and protect bacteria from environmental stress such as heavy metal toxicity and dehydration. When utilized in the bioremediation process, EPS should have anionic functional groups that are attached and either neutral or negatively charged so that it can operate as a workable biosorbent [70]. Both processes, *viz.,* biofilm formation and exopolysaccharide synthesis, are interlinked and required for the biomineralization and metal biosorption processes. A significant number of exudates, debris, and polysaccharides produced by one or more species are enclosed within the biofilm.

Sometimes the EPS can be distorted or altered through phosphorylation, carboxymethylation, methylation, and sulphonation, which helps the biological activity of the polymer and expands its application. Exopolysaccharide-producing microorganisms have a high concentration of anionic groups. They have been shown to have metal-ion chelation abilities, which will aid in removing dangerous metals from the environment [70]. Some commercially used microbial species for the EPS and biofilm-producing isolates are *Pseudomonas aeruginosa* and *Azotobacter vinelandii* for alginate*, Pseudomonas aeruginosa* for hyaluronan, *Sphingomonas paucimobilis* for gellan, and *Xanthomonas campest* for xanthan. Overall, the benefits of these microbes have a substantial impact on plant vitality. Traditional rhizoremediation and phytoremediation applications gain a new perspective from microbe-plant interactions that target metal stress. The interactions that lessen metal stressors include those produced by Plant Growth Promoting Bacteria (PGPB), organic acids, biosurfactants, biomethylation, redox processes, phosphate solubilization, nitrogen fixation, and iron sequestration [71]. These techniques support biomass production and phytoremediation. To effectively detoxify heavy metal contamination, a full study of hyper-accumulator plants and their interactions with microorganisms may be necessary.

### 3.1. Strategies for Reconstructed Metabolic Pathways in Bioremediation Techniques

**Figure 3:**
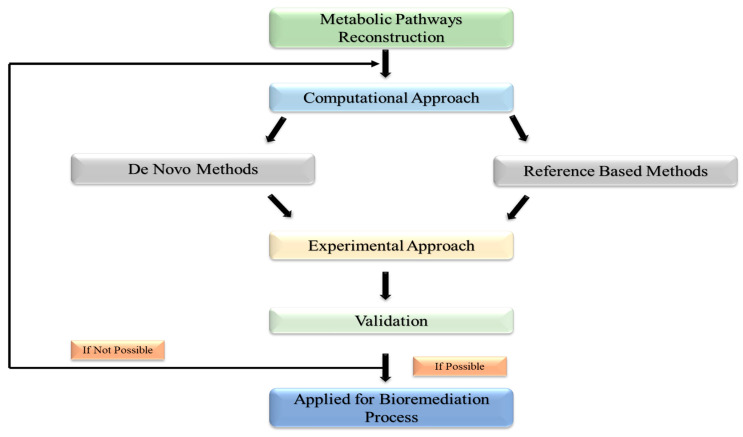
Depicting the experimental technique approach used for validation of *in silico*-designed pathways.

Using high-tech breakthroughs in WGS (whole-genome sequencing), directed evolution approaches, and high-throughput screening, decades of research have created a platform for reconstructing novel metabolic pathways for the bioremediation of different persistent contaminants in the environment [72]. In general, there are two methods for reconstructing metabolic pathways: (1) the in-silico method, which builds microbial pathways using a variety of tools and computational algorithms; and (2) the experimental method, as depicted in Figure 3, which validates in-silico-designed pathways using a variety of methods and molecular biology tools [73]. Computational techniques are used to reconstruct existing metabolic pathways [74]. The ability to quickly reconstruct pathways for the bioremediation of hazardous refractory substances is made possible by the availability of sizable databases connected to WGS (whole genome sequencing) and data from previously researched natural metabolic pathways. Through the use of this method, novel microbial pathways are created by assembling enzyme-encoding genes from various species. MetaCyc, KEGG, BRENDA, and Rhea are enzyme-catalyzed biochemical process databases that detail enzymes involved in constructing metabolic pathways with additional reactions [75].

These databases’ routes do not take into account the biology of the microbes that encode the pathways; instead, they just represent chemical activities. To compare metabolic models across species, one can use these reference pathways, also referred to as integrated pathways. Use the sequence alignment program BLAST to find statistically significant matches between query sequences (nucleotides or proteins) and sequence databases. This strategy is anticipated to produce functionally analogous proteins since it is predicated on the notion that homologous sequences in known and undiscovered networks encode functionally identical proteins. A sophisticated automated metabolic network called GSMM (Genome-Scale Metabolic Model) is also utilized to recreate bioremediation processes [76]. This genetics-based approach helps in the prediction of microbial phenotypes. GSMM constructs the network by utilizing software, data resources, and genetic information from specific microorganisms. KEGG and BioCyc, for example, provide organism-specific pathways for whole genomes, whereas MetCyc helps reconstruct all these mechanisms to produce specific metabolites [77]. MEGAN, KAAS, and MG-RAST tools aid in high-efficiency route reconstruction. Model SEED, which rebuilds metabolic networks in a table-like style utilizing functionally related genes coding for enzymes, and MEGAN, KAAS, and MG-RAST tools aid in high-efficiency route reconstruction. The MAPLE program analyzes vast volumes of metagenomics data in species dispersion investigations. Similarly, the COBRA (constraint-based reconstruction and analysis) tool is used to anticipate appropriate genetic alterations to optimize the rate and yield of metabolite synthesis [78].

### 3.2. De Novo Metabolic Route Reconstruction Using Computational Techniques

To develop bioremediation methods, metabolic pathway rebuilding by the *de novo* strategy utilizes the intrinsic variety of microorganisms through enzyme-based reactions. Predicting biological processes from metabolite chemical structure involves reconstructing metabolic pathways from scratch [79,80]. Two approaches make up the majority of this strategy. The first technique uses computer programs that take an unknown drug and automatically generate the chemical structures of the intermediate compounds along the anticipated metabolism pathway. The University of Minnesota’s PathPred and UMPPS systems offer free web servers for predicting various metabolic pathways. On the other hand, the second-category servers carry different identified chemical structures for the metabolic framework. This approach is widely used due to the known structure of chemical compounds and the previously stored data in all databases. In terms of computing costs, this technique has a limitation. Furthermore, it is ineffective for forecasting multiple chemical pathways simultaneously. The retro-biosynthesis process is also used for pathway construction by modifying the target molecules using chemical transformation principles. The pathway prediction system (PPS) has been developed for the biodegradation of different xenobiotic compounds in the environment. It has a user-friendly interface that allows a specific selection of the desired reaction. PathPred, an efficient prediction system, is concerned with several plants’ xenobiotic compound biodegradation and secondary metabolite synthesis [81]. Supervised learning has been recently described as a one-of-a-kind computationally efficient technique. It can predict enzyme-catalyzed compound reactions. This method can handle thousands of metabolites at the same time [82].

## 4. Nanoparticles and Their Role in Heavy Metal Bioremediation

### 4.1. Nanoparticles

Both nature and science have long used nanoparticles. They serve as a bridge between bulk materials and molecular structures, which has made them of tremendous interest. They exhibit quantum effects while being so tiny. They have improved stability, strength, and reactivity in addition to having surprising optical features, all of which make them very important. They have been employed for a long time in a variety of industries, including cosmetics, the manufacture of iridescent glassware, the manufacture of weaponry, paints, pharmaceuticals, Roman pottery, textiles, and many others. Nanoremediation is the practice of using nanotechnology in remediation procedures. When compared to bulk materials, nanoparticles are atomic or molecular aggregates with sizes between 1 and 100 nm that might alter their physiochemical properties. Its classification, such as 0-dimensional and 1-dimensional nanoparticles, depends on how many dimensions electrons may be housed in [83]. They have special qualities that differentiate them from their bulk equivalents and give them a wider range of uses. Due to their small size, materials with a high surface area-to-volume ratio have unique physical and chemical properties. Both organic and inorganic nanoparticles (micelles, fullerenes, and dendrimers) are present (ceramic, steel, and metal oxide nanoparticles). Although nanoparticles can be polycrystalline or amorphous and have a variety of morphologies, including platelets, spheres, and cubes, nanocrystals are single-crystalline nanoparticles. Both chemical and biological processes have been used to create nanoparticles. The biological synthesis method is popular due to its low cost, quick synthesis, control over size and features, and toxicity. Self-organization and the production of molecules with highly selective properties are capabilities of biological systems. The physical properties of nanoparticles are influenced by size, shape, distribution, surface area, solubility, and structure. The surface area to volume ratio of nanoparticles increases exponentially as the number of molecules at the surface increases, making the surface more reactive [84].

Furthermore, as the nanoparticles size and shape change, so do their optical properties. The zeta potential, surface chemistry, photocatalytic capabilities, and chemical composition of nanoparticles define their chemical properties [85]. Green nanotechnology creates nanoparticles from living organisms such as bacteria and plants. Microbes have sparked interest in the production of nanoparticles due to their high tolerance, rapid purification, and reproducibility. It has been demonstrated that biologically produced nanoparticles have excellent catalytic reactivity and a specific surface area [86]. A capping agent delivered by microorganisms aids in preventing nanoparticle agglomeration. Extracellular nanoparticle production requires no downstream processing and is inexpensive [87].

### 4.2. As Carriers for the Active Component during Bioremediation

An innovative technology that can be applied to the subject of environmental bioremediation is the combination of enzymes with nanomaterials. Nanomaterials, which are regarded as particularly fascinating matrices because of their distinctive physicochemical features, can be successfully used to immobilize a variety of physiologically active compounds. As a result of their potential to form nanobiocatalysts, nanoparticles are carriers that have undergone much research. Nanographene, nanotubes, nanofibers, and nanogels are only a few examples of innovative hybrid nanocomposites that are currently being developed. Many problems were exposed by heavy metal contamination of arable soil, including the phytotoxic effects of several elements, including Cd, Pb, Zn, and Cu. These are all well-known essential metals, but after the critical endogenous levels are surpassed, they lead to a number of phytotoxicities [88]. As a result, HMs are poisonous and regarded as environmental pollutants, bioremediation might be a good choice to treat contaminated areas. Although bioremediation is a great way to remove different kinds of pollution, it has significant drawbacks. For instance, bioremediation could not be effective in locations with high concentrations of harmful contaminants. It involves HMs and their salts [89]. Furthermore, as living standards have increased due to scientific and technological advancements, hazardous waste has increased. As a result, cleaning up the ecosystem by eliminating toxins with present technology is inefficient and useless. Living things have evolved to flourish in metal-rich surroundings by utilizing a variety of coping mechanisms. These procedures may entail modifying the harmful metal’s properties, rendering it less toxic, and producing pertinent metal nanoparticles. As a result, the production of nanoparticles is seen as a “by-product” of a resistance mechanism against a particular metal and can be used as a substitute method for doing so.

Understanding the behavior of nanoparticles requires knowledge of their morphology, particle size distribution, specific surface area, surface charge, and crystallographic characterization. For a variety of reasons, different nanomaterials (NMs) are used in bioremediation. For instance, when the matter is scaled down to the nanoscale, a material’s surface area per unit mass rises; as a result, more of the substance can come into contact with other components, which might alter the reactivity. Less activation energy is needed to facilitate chemical reactions because NMs have a quantum impact. A novel method called nanobioremediation is showing promise in several industries. Microorganisms are increasingly being used in the production of nanoparticles as nanofactories and as possible tools for environmental cleanup [90]. Nanoparticles and nanomaterials created by bacteria utilizing nanotechnologies are used in nanobioremediation to remove environmental pollutants from polluted locations, including HMs and organic and inorganic pollutants. Microbes, flora, and enzymatic remediation are the three main bioremediation techniques [91]. Jiamjitrpanich et al. [92] found that using *Panicum maximum* in nano-phytoremediation was a more efficient way to contaminate and remove contaminated soil. Magnetic nanoparticles have numerous uses in adsorption and catalytic pollution remediation, according to Ajmal et al. [93].

Immobilizing pollutants on-site has emerged as a practical and affordable technique for cleaning up contaminated soils. To identify a suitable material that takes into account low cost, high efficiency, greater stability, the least detrimental environmental effect, and maximal performance, several NM-based full-form modifications have been examined. Moreover, HMs from water and organic and inorganic contaminants from soil can be eliminated by nanoparticles. For instance, organochlorines and long-chain hydrocarbons are very resistant to microbial and plant breakdown [94]. The bioremediation method using several nanoparticle methods is shown in Table 2.

### 4.3. Nanomaterials as Active Additives for Bioremediation

Numerous shortcomings of traditional remediation techniques have been overcome thanks to the application of nanotechnology. Utilizing biogenic nanoparticles or materials made from biological sources, nanobioremediation is an extension of nanotechnology that deals with the removal of pollutants from the contaminated area. Due to the size of the material, this process has an advantage over other ways since a smaller size would result in a larger surface area to volume ratio, which would open up more surface area for the reaction to take place. The environmentally and economically beneficial characteristics of green nanoparticles as additives for bioremediation have attracted a lot of interest recently. Nanoparticles and phytoremediation can be combined in enzyme-based bioremediation. Integrating nanotechnology and biotechnology would allow for the quick degradation of these substances by bacteria and plants. Nano-encapsulated enzymes would break down complex organic compounds into simpler ones. Bacteria may mobilize and immobilize metals, and in some situations, microorganisms that can decrease metal ions can precipitate metals at the nanometer scale [113]. Bacteria are also being studied as a possible “bio-factory” for producing nanoparticles such as gold, silver, platinum, palladium, titanium, titanium dioxide, magnetite, cadmium sulfide, and others [114]. According to Alao et al. [115], zerovalent nanoparticles can easily remove various metallic contaminants from soil and waste-water effluent. Several halogenated hydrocarbons, organic compounds, and radionuclides have also been remedied using nanoparticles. For Pb(II) and Cr, the degradation degree of nanoscale zerovalent iron is 30 times greater than that of iron powder (VI). In degrading arsenic forms (As (V) and As (III)), the degradation rate of nano-adsorbent iron oxide is 8–10 times faster than that of the micron scale. Filtering is a successful method for purifying nanoparticles further. Bacterial cells and surface layers have distinct metal-binding properties, making them useful in bioremediation and nanotechnology applications [115]. Bioremediation by microorganisms typically requires using known aerobic and anaerobic bacteria to remove pesticides and hydrocarbons. Rhizoremediation is a low-cost and successful method of cleaning polluted soils using the joint action of plants and their symbiotic bacteria in the rhizosphere.

Studies have been conducted on the use of NPs in the bioremediation of heavy metal-contaminated sites. It has been reported that they can relieve drought stress and reduce Cd toxicity in wheat plants by enhancing biomass, chlorophyll content, and antioxidant biocatalysis. Si NPs have been reported to reduce HM-induced phytotoxicity in wheat, rice, and peas. To lessen the detrimental effects of HMs on plant growth and development, new nanoremediation techniques must be developed [116]. Bacterial nanoparticles can bind to and concentrate dissolved metals and metalloid ions. They can convert toxic metal ions into non-toxic nanoparticles. Bacterial mobilization, immobilization, and metal precipitation all contribute to nanoparticle formation.

The versatility and diversity of bacteria-produced nanoparticles make it a viable strategy [117]. Bacteria detoxify their immediate cell environment by converting toxic metal species into metal nanoparticles. Bacterial biomolecules are used as stabilizing and capping agents in the production of nanoparticles. Extracellular synthesis of biogenic nanoparticles is more efficient and produces easier-to-remove nanoparticles. Extracellular synthesis of large quantities of nanoparticles is possible. Bacteria have been used to produce nanoparticles such as palladium, titanium, magnetite, gold, and silver. Bacteria have the potential to be used as a biocatalyst for inorganic material synthesis, a bioscaffold for mineralization, and an active participant in nanoparticle production. Biosynthesis using bacteria is a versatile, reasonable, and acceptable large-scale production technology [118]. It has been reported that biogenic manganese oxide nanoparticles produced by *Pseudomonas putida*, silver nanoparticles produced by *Bacillus cereus*, gold nanoparticles produced by *Rhodopseudomonas,* and biogenic selenium nanoparticles produced by *Citrobacter freundii* Y9 performed the best bioremediation [119,120,121,122].

As a result, nanotechnology greatly improves the process of bioremediation, and its application in heavy metal bioremediation has been widely exploited. Controlling, sensing, and remediating pollutants with nanoparticles are some approaches used to monitor and treat contaminants. Chatterjee et al. (2019) created myco-synthesized iron oxide nanoparticles to remove HMs from waste water. The extracellular synthesis of nanoparticles with *Aspergillus niger* BSC-1, a mangrove fungus, resulted in the successful synthesis of biogenic (fungus) nanoparticles in the form of nanoflakes (20–40 nm) that could remove chromium through adsorption with excellent efficiency at a specific pH and temperature [123]. Keskin et al. [98] developed effective *Lysinibacillus* sp.-encapsulated nanofibers with cyclodextrin for hexavalent chromium, nickel, and dye remediation. These nanofibers functioned as a carrier matrix and a food source for the encased bacterium. In the presence of a reducing biomolecule, magnetic iron nanoparticles were produced in a living *D. radiodurans* R1 strain and demonstrated remarkable arsenic removal capacity [124]. Subramaniyam et al. [125] successfully produced iron nanoparticles from *Chlorococcum* sp. MM11 can remediate and reduce 92% of hexavalent chromium to trivalent chromium. Mukherjee et al. [126] developed aloe vera-based biogenic nanoparticles. This environmentally friendly method has been demonstrated to be highly effective in removing arsenic from contaminated water [126]. Another study by Al-Qahtani demonstrated that zero-valent silver nanoparticles derived from *Ficus benjamina* leaf extract efficiently removed cadmium [127]. It was found that the initial metal ion concentration influenced contaminant clearance and that a color shift identified the creation of silver nanoparticles as brown [128,129]. Different types of PAHs and HMs can be dealt with by certain types of bacteria and fungi that are present in the environment concurrently or successively [130]. Although native to HMs-contaminated sites, filamentous fungi have significant bioremediation potential that is frequently untapped [131]. One of the largest gene pools of invertebrates, bacteria, fungi, algae, and protozoa can be found in soil [132]. A more effective and broad-spectrum breakdown of pollutants is made possible by engineering competent microbes to enhance cell membrane transport or enzymatic characteristics. Future bioremediation will be more effective and last longer because of modifications and adaptations made to nanotechnology.

## 5. Future Challenges

Using heavy metal-tolerant microorganisms in conjunction with their host plants can be an environmentally safe and cost-effective strategy for treating HM-polluted soils. Though there is currently a lack of information to commercialize this technology. When heavily polluted areas are contaminated, the lodgement of metals in plant parts frequently slows the clean-up process. It has been shown that HMT-PGP microorganisms with supplements (nutrients) are more effective in polluted soil. In microcosm-scale phytoextraction studies, adding thiosulfate products to metal-tolerant bacteria increased Ar and Hg mobilization and absorption by *L. albus* and *B. juncea*. Microbes genetically engineered to be well suited to different biogeographical conditions can also effectively remove HM from contaminated soils. The addition of nutrients can also encourage the local microbial community and cause the soils that have been contaminated with HM to heal and detoxify. The ability of HMT bacteria in consortia to remove HM from contaminated environments has recently been put to the test. Entomopathogenic fungi can be used to eradicate HM from contaminated soils. This can also be applied to contaminated and infected soils for biocontrol and cleanup. For better HM removal, microbes and plants can be created or modified via engineering routes. The genetically designed microbial sensors have demonstrated a speedy detection approach for improving polluted soil with exact assessment and are also seen as a viable idea. According to recent research, phytoremediation methods for heavy metal bioremediation may be improved by examining plant microbiomes in contaminated soils.

## 6. Conclusions

The rapid expansion of agriculture and industry over the last several decades has contaminated the environment with various hazardous wastes, including plastics, HMs, chlorinated biphenyls, and agrochemicals. Using plant-microbe synergy to repair polluted soils is a promising but experimental method. This study explains the utility of microbes as a superior method for removing heavy metal detoxification from polluted areas over biophysical techniques, which are less effective and costlier due to the amount of energy expended. PGP microorganisms use precipitation, biosorption, enzymatic metal transformation, complexation, and phytoremediation methods. HMT-PGP microorganisms have several advantages, including enhanced soil quality, the removal of toxic compounds, increased plant development, and HM removal from soil. However, it is necessary to develop appropriate bio-formulations for the remediation and use of polluted soils using HMT-PGP microorganisms. Extending our understanding of the nanotechnology microbes to improve and digest the contaminants and performing field studies would undoubtedly pave the way for advancements in this sector. This low-input, sustainable application can potentially extract HMs from contaminated soil and improve the quality and productivity of the soil. Therefore, microorganisms provide a valuable platform that may be employed to enhance the bioremediation model for different environmental contaminants to manage environmental pollution.

## Figures and Tables

**Table 1:** Bioremediation processes facilitated by different PGP microbes and associated mechanisms.

Microbes	Plant	Isolated Sources	PGP Traits	Metal Stress	Experimental Conditions	Results	References
*Sinorhizobium meliloti*	*Medicagolupulina*	Root nodule of *M. lupulina*	Siderophore production, IAA, and ACC deaminase activity	200 mg kg^−1^ Cu	1.6 mM Cu	Enhanced Cu uptake and improved plant growth. Antioxidant activity in excess Cu stress conditions	[30]
*Rhizobium halophytocola*	*Ciceraritenum*	Root nodule	IAA and phosphate solubilization	4 mM Pb	1 Mm Ni	Increased plant growth and yield. Reduced as plants absorb	[31]
*Mesorhizobium* sp*. RC4*	*Helianthus annuus*	Contaminated soil with Cr	P solubilization and IAA production	16 mM Ni	5 mM Cr	Enhanced Ni and Cr uptake and plant growth	[32]
*Bradyrhizobium*	*Brassica juncea*	Cr and Zn hyperaccumulators in stems		1.8 mM Zn	1.4 mM Cr	Increased shoot and root length	[33]
*Rahnella*	*Brassica napus*	Roots of *Polygonumpubescens* with Mnhyperaccumulator region	Phosphate solubilization, IAA, and siderophore production	200 mg kg^−1^ Zn, 25 mg kg^−1^ Cd,	150 mg L^−1^ Cu, 1550 mg L^−1^ Cd, and 3000 mg L^−1^ Zn	Increased uptake levels of Zn, Pb, and Cd in the aerial parts and root tissues of plants.	[34]
*Acinetobacter*	*Ciceraritenum*	Contaminated soil with As	IAA, ACC deaminase activity, and phosphate solubilization	10 mg kg^−1^Arsenic (V)	1500 mg L^−1^ As (III)	Increased plant yield and growth. Reduced As uptake by plants	[35]
*Bacillus pumilus*	*Sedum plumbizincicola*	Cd and Zn hyperaccumulators in stems	Siderophore production, IAA, and ACC deaminase activity	5.9 mg kg^−1^ Cd, 153 mg kg^−1^ Pb	400 mg L^−1^ Cd, 3500 mg L^−1^ P	Increased plant biomass and Cd uptake by colonization in the rhizosphere	[36]
*Rhodococcuserythropolis*	*Sedum plumbizincicola*	Roots of *S. Graptosedum*	P solubilization and IAA production	132 mg kg^−1^ Zn	20 mg L^−1^ Cd, 600 mg L^−1^ Zn	Enhanced Cd uptake and plant growth	[37]
*Pseudomonas* sp.	*Helianthus annuus*	Torch lake sediment	Phosphate solubilization, IAA production	500 mg kg^−1^ Cu	6 mM Zn, 5 mM Pb, 0.3 Hg	Increased Cu and Cd accumulation in sunflowers. Increased total biomass of plants	[38]
*Klebsiella* sp.	*Triticum aestivum*	Rhizospheric soil of maize with industrial effluent	ACC deaminase, exopolysaccharide, oxidase, siderophores, and IAA production	80 mg kg^−1^ Cd	500 mg L^−1^ Cd	Lowered Cd uptake and promoted wheat growth	[39]
*Bacillus* sp.	*Brassica juncea*	*Alnus firma* roots	IAA and siderophores production	150 mg L^−1^ Pb	150 ppm Cd, 150 ppm Ni, and 800 ppm Cu	Increased shoot and root length	[40]
*Microbacterium*	*Salix caprea*	Plant tissues of *S. caprea*	Siderophore production, IAA, and ACC deaminase activity	608.2 mg kg^−1^ Zn, 98.5 mg kg^−1^ Pb	4 mM Cd	By increasing the concentrations of Zn and Cd in leaves	[41]
*Rhizobium* sp.	*Lens culinaris*	Root nodules of lentil plants	ACC deaminase activity, phosphate solubilization, HCN, and ammonia production	290 and 580 mg kg^−1^ Ni	300μgmL^−1^ Cd, 1400μgmL^−1^ Pb	Decreased uptake of Ni, increased nodulation, nitrogen content, chlorophyll, and seed yield	[42]
*Rhizobium, Pseudomonas, Arthrobacter, Agrobacterium, and Serratia*	*Brassica juncea*	Root nodules of *Brassica*	Ammonia production, IAA, and siderophores production	80 mg kg^−1^ Cd	50 mg L^− 1^ ZVI-NPs	Enhanced toxic metal uptake and improved plant growth	[43]
*Arthrobacter and Enterobacter*	Mustard	Plant tissues of sunflower	By ACCD, phytohormone, siderophore, and dissolving insoluble mineral nutrients	190 mg kg^−1^ Ni	141.34 mg g^−1^ of Ni and Cd	By increasing the concentrations of heavy toxic metals in leaves	[44]
*Actinobacteria and Mycobacterium*	*Triticum aestivum*	From rhizospheric soil	IAA, siderophore production, and ACC deaminase activity	180 mg kg^−1^ Cu, Ni, and Cd	151.34 mg g^−1^ of Ni and Cd	By enhancement of Cd and Cu uptake	[45]
*Rhodotorula mucilaginosa*	*Brassica juncea*	Root nodules of *Brassica*	Ammonia production, IAA, and siderophores production	156 mg L^−1^ Cr	4.79 to 10.25% for planktonic cells	By biofilm formation	[46]
*Arthrobacter, Azoarcus, Alcaligenes, and Enterobacter*	Sunflower and mustard	Plant tissues of sunflower	By ACCD, phytohormone, siderophore, and dissolving insoluble mineral nutrients	290 mg kg^−1^ Ni	141.34 mg g^−1^ of Ni and Cd	By increasing the concentrations of Ni and Cd in leaves	[47]
*Ralstonia eutropha*	Indian mustard, alfalfa, and sunflower	From rhizospheric soil	IAA, siderophore production, and ACC deaminase activity	200 mg kg^−1^ Cu	800 ppm Cu	Enhanced Co and Cu uptake and improved plant growth	[48]

**Table 2:** Bioremediation approach using different mechanisms of nanotechnology.

Associated Microbes	Modification	Applied Nanotechnology	Mechanism	Removal Capacity	References
Actinomycetes	Synthesized organic framework in actinomycetes	Silica nanomaterials	Degradation by photocatalysis	By decolorization of industrial effluent (melanoidins and textile dyes), up to 80%	[95]
*Pleurotus ostreatus*	Immobilization of Laccase	Enzyme immobilization	Oxidation by laccase	Degradation of carbamazepine is 10–15%, and bisphenol-A degradation is 90%	[96]
*Synechococcus*	Sol-gel method	By optical biosensor	By detection of heavy metal	Cd^2+^ and Cr^6+^	[97]
*Lysinibacillus*	Encapsulation by bacterial cells	By cyclodextrin fibers	By bacterial remediation	Removal efficiency of Cr(VI) = 58 ± 1.4% and Ni(II) = 70 ± 0.2%	[98]
*Pseudomonas aeruginosa*	Encapsulation by bacterial cells	By spun nanofibrous webs	By removal of different dyes	Removal of methylene blue up to 55–70% at polymer matrices (polyvinyl alcohol and polyethylene oxide)	[99]
*Pseudomonas aeruginosa*	Synthesized from bacterial cell-free culture supernatant	By Zirconia nanomaterial	By electrostatic interaction among zwitter ions	Tetracycline adsorption of 626.67 mg/g	[100]
*Aspergillus nidulans*	Modified activated carbon	By enzyme immobilization	By bacterial remediation		[101]
*Chlorella vulgaris*	Enzyme immobilization	CeO2 nanoparticles	By detection of heavy metal	The precursors used to create the final products have defensive qualities. The cells that were immobilized were shielded from UV and H_2_O_2_.	[102]
*Saccharomyces cerevisiae*	Immobilization of bacterial cells	Sol gel method	By examining the thickness of the generated films and the shape of the resulting structures.	By bacterial remediation	[103]
*Rhodococcus ruber*	Immobilization	Aluminosilicate	Investigate the structure, mechanical properties, and biological activity of ceramic composites.	By degrading phenol by 10%	[104]
*Pseudomonas* and *Arthrobacter*	By electrostatic interaction among zwitter ions	By spun nanofibrous network	By bacterial remediation	By decolorization of industrial effluent, up to 65%	[105]
*Arthrobacter* and *Methosinus*	By genetically engineered bacteria	By metal mobilization	Microbe-assisted phytoremediation	Removal of polycyclic hydrocarbons (PAHs) on a large scale	[106]
*Penicillium sp*	By extracellular sequestration	Precipitation and adsorption	By detoxification	detoxify Hg(II) ions by 5–7%	[107]
*Micrococcus, Enterobacter, and Flavobacterium*	By metal absorption process	By biofilm bioremediation	By biosorption	4.79 to 10.25% of toxic metals	[108]
*Staphylococcus epidermidis*	Microbe-assisted phytoremediation	Enzyme immobilization	By carbonate mineralization	86% Pband 76.8% Cr(VI)	[109]
*Aspergillus sp.* and *Rhizopus sp.*	By electrostatic interaction among zwitter ions	By mobilization of metals	By bioaccumulation and metal leaching processes	Removal of 6–8% of the dry cell mass	[110]
*Agrobacterium*	Encapsulated in alginate with iron oxide nanoparticles	By nanoparticles	By adsorption process	197.02 mg/g for Pb	[111]
*Aspergillus tamarii* and *Aspergillus ustus*	Microbe-assisted phytoremediation	By mobilization of metals	By fungal remediation	58.6% and 80% for chromium and arsenic, respectively.	[112]

## Data Availability

Not applicable.
